# Endogenous Bufadienolides, Fibrosis and Preeclampsia

**DOI:** 10.1155/2019/5019287

**Published:** 2019-12-12

**Authors:** Vitaliy A. Reznik, Vladimir A. Kashkin, Natalia I. Agalakova, C. David Adair, Alexei Y. Bagrov

**Affiliations:** ^1^Department of Obstetrics and Gynecology, School of Pediatric Medicine, St. Petersburg 194353, Russia; ^2^Valdman Institute of Pharmacology, Pavlov Medical University, St. Petersburg 197022, Russia; ^3^Sechenov Institute of Evolutionary Physiology and Biochemistry, 44 Torez Prospect, St. Petersburg 194223, Russia; ^4^Department of Obstetrics and Gynecology, University of Tennessee, Chattanooga, TN, USA

## Abstract

Frequency of preeclampsia has no tendency to decrease, and it still takes the leading position in the structure of maternal mortality and morbidity worldwide. In this review, we present the “fibrotic concept” of the etiology and pathogenesis of preeclampsia which involves system consisting of Na/K-ATPase and its endogenous ligands including marinobufagenin. New therapy of preeclampsia includes modulation of the Na/K-ATPase system by immunoneutralization of the marinobufagenin and use of mineralocorticoid antagonists which are capable to impair marinobufagenin-Na/K-ATPase interactions.

## 1. Introduction

Hypertension is the most common medical condition encountered during pregnancy, with a frequency of approximately 6–8% [1]. The hypertensive disorders of pregnancy cover a spectrum of conditions, including preeclampsia-eclampsia, gestational hypertension, and chronic hypertension. Working Group on High Blood Pressure in Pregnancy defines hypertension in pregnancy as two blood pressure measurements of ≥140/90 mmHg measured≥six hours apart [1]. Despite ongoing prevention and repeated attempts to select a therapy, it was not possible to reduce the frequency of preeclampsia (PE). The diagnosis of PE is made by the combination of arterial hypertension (systolic blood pressure ≥ 140 mmHg and(or) diastolic blood pressure ≥ 90 mmHg [2]), proteinuria (presence of protein in the urine ≥ 0.3 g/l in the daily sample (24 h) or in two samples taken at intervals of 6 h; using the test strip (protein in the urine) [2], the indicator (0.3 g/l [3])), and generalized edema. Peripheral edema is observed in 70–80% of cases with physiological pregnancy, therefore, cannot be considered as a diagnostic criterion for PE [4].

The mechanisms implicated in the pathophysiology of PE include oxidative stress, placental steroid formation, and insulin resistance [5, 6]. The endpoint of this complex interplay between maternal and placental mechanisms is a maternal multisystem disorder, characterized by hypertension, proteinuria, and multiorgan dysfunction. During PE, the placental spiral arteries fail to lose their elastic lamellae ultimately leading to decreased placental perfusion [7]. Placental hypoxia is frequently viewed as an early trigger of placental production of soluble factors resulting in endothelial dysfunction [8], which may play the main role in the pathogenesis of PE.

## 2. Fibrosis and Preeclampsia

The first observation suggesting that fibrosis can underlie the development of PE is related to the works of Tenney and Montgomery in 1935–1936. It has been shown that collagen fibers in the villous stroma are first visible on light microscopy as early as the 2^nd^ month of gestation. The authors found that the collagen content of the villi showed no appreciable increase between midpregnancy and midterm [9]. In 1968, Fox confirmed empirically the results of these works and suggested that placental fibrosis is not associated with any excess of fetal hypoxia complications. It is suggested that villous fibrosis is a result of a reduced fetal villous blood flow; such a reduction may be due, as in toxemia or diabetes, to obliterating endarteritis changes in the fetal stem arteries or, as in prolonged pregnancies, to a constriction of villous capillaries [9].

In later works, it was shown that women who have experienced PE are at increased risk of hypertension, coronary artery disease, and fatal stroke. Its effects do not end with childbirth and is associated with a higher cardiovascular risk lifelong. This increased risk could be partly explained by left ventricular systolic and diastolic impairment, arterial stiffening, and endothelial dysfunction which have been described during and after PE. These phenomena, which influence each other, are related to myocardial and vascular remodeling, a process leading to loss of function and fibrotic tissue deposition [10–12]. In early studies, it was suggested that the modified placental production of vasoactive substances appears to underlie the pathological mechanism of hypertension and vascular dysfunction in PE [13]. Over the past 20 years, it has been shown that endogenous digitalis-like factors or cardiotonic steroids (CS) of the Na/K-ATPase (NKA) are also among these substances, and elevated plasma levels of CS were detected in patients with PE [14–16].

**Figure 1:**
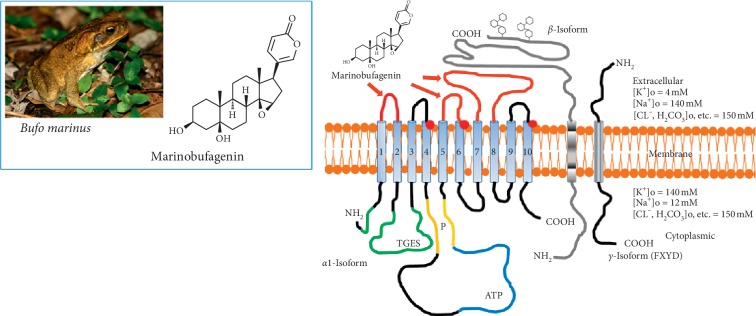
Chemical structures of bufadienolide, marinobufagenin and its pharmacological target, Na/K-ATPase.

Recently, it has been demonstrated that nanomolar concentrations of bufadienolide CS, marinobufagenin (MBG) (Figure 1) stimulate the synthesis of collagen and induce fibrosis in cardiovascular tissues and in the kidney [17]. One of the mechanisms underlying the profibrotic effect of MBG is EGFR-Src-dependent inhibition of Fli-1, a nuclear transcription factor and a negative collagen-1 regulator, causing its reduction which resulted in activation of collagen-1 [15]. During the experiments, it was shown that CS (MBG, ouabain, and digoxin) cause an increase in the number of embedded proline residues and also increase collagen synthesis in the fibroblast culture, which was evidence of the presence of signaling function of the Na/K-ATPase-CS complex with activation of Src and MAPK [17, 18]. *In vivo*, the administration of MBG in a concentration that is observed in renal failure caused the development of cardiac fibrosis with activation of the signaling pathway mediated by NKA, which was confirmed by an increase in Src and phosphorylation of a mitogen-activated protein kinase (MAPK) in the myocardium [17, 18].

Our observations confirmed and extended previously obtained findings demonstrating substantial elevations of MBG levels in PE. In our previous study, in patients with severe PE (161/104 mm Hg), plasma MBG concentration comprised averaged 2.63 nmol/L. Later, it was shown that in subjects with mild PE and lower arterial pressure, the mean level of MBG in plasma was 1.68 nmol/L [19]. Since plasma concentrations of MBG in subjects with noncomplicated pregnancy in these previous studies were similar and comprised 0.80 nmol/L and 0.63 nmol/L, respectively, the levels of MBG may be one of the markers of severity of PE. Our findings also indicate that MBG may not only be a marker but also a factor contributing to the pathogenesis of PE. In the “pathophysiologically relevant” range of concentrations (1–3 nmol/L), *in vitro*, MBG exhibits a 25% inhibition of NKA in the human vascular sarcolemma and induces a contractile response in the isolated rings of human mesenteric arteries [19]. Thus, the *in vivo* levels of MBG observed in patients with PE in our previous and present studies are capable of enhancing vascular tone and substantially inhibiting NKA.

**Figure 2:**
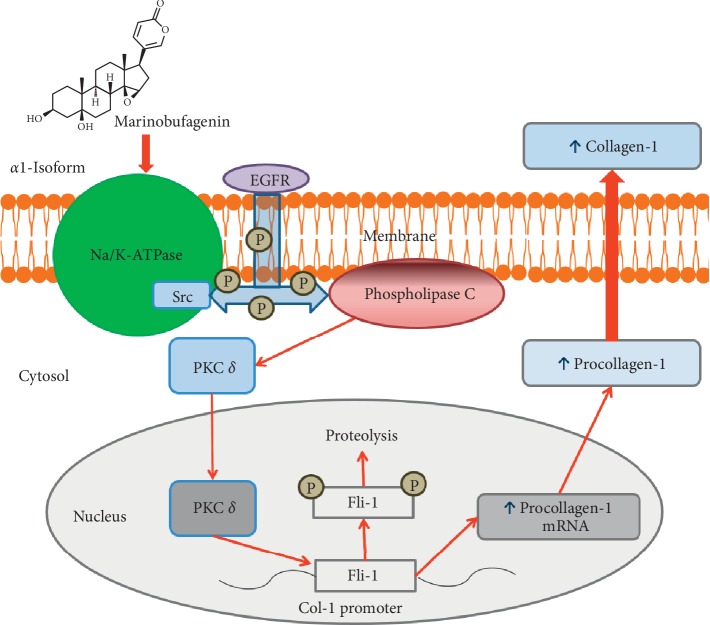
Scheme of the Fli-1-dependent profibrotic pathway activated via binding of marinobufagenin (MBG) to Na/K-ATPase (NKA). The interaction of MBG with NKA leads to activation/phosphorylation of Src followed by activation/phosphorylation PLC and PKC via scaffolding function of NKA.

The mechanism of PE includes remodeling of uterine spiral arteries and making them fibrotic by a variety of vascular factors including cardiotonic steroids [20, 21]. Physiological function of CS is regulation of sodium excretion by means of inhibition of NKA in the epithelial cells of renal tubuli, i.e., an adaptive response [20]. Under pathological conditions, for example, in patients with PE, side effect of this primarily adaptive response becomes essential, and CS cause inhibition of sodium pumps in tissues including vessels [22]. Recent studies demonstrated that in addition to vasoconstrictor effect of CS, these compounds directly affect intracellular signaling resulting in the loss of vascular elasticity and vascular fibrosis [23]. Low concentrations of CS acting on PKC delta induce deactivation of Fli-1 and cause expression of collagen-1, which is a key factor of fibrosis in blood vessels, myocardium, and kidney [23, 24]. Recent studies demonstrate the clinical importance of fibrosis in the cardiovascular system and placenta [25, 26], and moreover, suggest that blood vessels exposed to PE environment exhibit greater responsiveness to injury despite resolution of PE [27]. The general scheme of the development of MBG-induced fibrosis is presented in Figure 2.

## 3. Treatment of Preeclampsia

For millennia, the venomous secretions of amphibians, which contain a large number of bufadienolide derivatives, are widely used in the traditional medicine of different civilizations. One of the most well-known examples from the traditional Chinese medicine is *Sen-So* (or *Ch`an Su*), which was used for the treatment of heart failure [28]. Being highly active substances, bufadienolides often caused toxic effects. Human fatal poisonings from toad venom have been reported in the many countries by the venoms from the Colorado River toad (*Bufo alvarius*), the cane toad (*Bufo marinus*), and the Chinese toad (*Bufo gargarizans*) [29]. Over the past 20 years, the digoxin-specific Fab fragments (Digibind) have been successfully used in the poisoning of toad venom, and it has shown their effectiveness in animals [30] and humans [31]. It is well known also that in patients at the final stage of renal failure [32] or PE [33], as well as in Dahl-S (salt sensitive) rats with salt load [34, 35], the erythrocyte NKA is inhibited, which can be reversed incubation, *ex vivo* with antibodies to MBG or with Digibind, and bovine digoxin antibodies that interact with CS [33, 36].

## 4. Monoclonal Antibody

Immunoneutralization of the CS in patients with PE is not new. The similarity of the functional profile (inhibition of NKA) and the structure of cardenolides (digoxin and ouabain) and bufadienolides (MBG and resibufogenin) allowed clinical use of polyclonal antibodies against digoxin (Digibind) in the treatment of PE [37, 38]. Digibind and DigiFab have been used for many years to treat digoxin intoxication [39]. Digibind has been shown to induce a decrease in blood pressure in animals with volume-expanded hypertension by an interaction between Digibind and an endogenous digoxin-like factor [39]. Digibind demonstrates cross-reactivity against the bufadienolides and cardenolides [19]. In a rat model of PE, the Digibind treatment produced lowering of the blood pressure and proteinuria [40]. In a clinical study, it was shown that antibody therapy by Digibind prevents a decline in renal function with well tolerated in severe PE by neutralizing CS [37].

Based on the results of previous studies, the involvement of MBG was demonstrated in the development of fibrosis. This mechanism was also shown on the PE model [41]. In this study, the umbilical arteries, obtained from pregnant women suffering from PE, contained greater amounts of collagen, less Fli-1, and they also showed low sensitivity to the vasorelaxant effect of sodium nitroprusside. Our laboratory demonstrated that in PE plasma levels of MBG increase and that *ex vivo* anti-MBG antibody reverses PE-induced inhibition of erythrocyte NKA [19, 33]. In pregnant NaCl-supplemented rats, increased MBG levels accompanied the development of symptoms of PE including elevation of arterial pressure, proteinuria, and reduction of fetal weight and size [33, 42]. In this model, *in vivo* immunoneutralization of MBG with poly- and monoclonal antibodies produced antihypertensive effect associated with the increase in vascular NKA activity [33, 42]. Considering that, (i) MBG stimulates synthesis of collagen, (ii) fibrosis in placenta and umbilical arteries in PE patients is accompanied by elevated placental MBG and a dramatic decrease in Fli-1 level [43], and (iii) PE is associated with vascular stiffening [44, 45]; it has been showed that MBG is one of the factors, implicated in pathogenesis of PE via induction of vasoconstriction and vascular fibrosis [41]. In the recent study, it has been shown that the placental levels of Fli-1 were dramatically lower, while collagen-1 levels were higher in both placenta and umbilical arteries in PreE samples compared to the tissues from control noncomplicated pregnancy. The subsequent treatment of the umbilical artery explants from PE patients with anti-MBG mAb was accompanied by a significant decrease in collagen-1 abundance [43].

Furthermore, in comparative study of the efficacy of specific antibodies with antidigoxin (DigiFab) and monoclonal antibody against MBG (3E9), it was shown that monoclonal antibodies restore the activity of PE-inhibited NKA in erythrocytes better than DigiFab, which suggested the involvement of MBG in the pathogenesis of PE [46]. In a recent study, it was shown that the administration of *humanized monoclonal antibodies* (206–208, H1L2) against MBG attenuate MBG-induced downregulated PCNA and upregulated p38 phosphorylation in cytotrophoblast cells, indicating a possible therapeutic action in PE [47]. Therefore, immunoneutralization of MBG is a logical step in the treatment of PE.

## 5. Mineralocorticoid Antagonists

The fact that mineralocorticoid antagonists can offset the effects of digitoxin in rats has been first reported by Selye [48, 49]. Subsequently, spironolactone and its active metabolite, canrenone, were reported to reverse digitalis toxicity and to lower blood pressure in rat hypertension models, in which levels of CTS are elevated [50–52]. Most recently, spironolactone was reported to suppress cardiac fibrosis in rats chronically treated by MBG [53]. Notably, in this study, MBG exhibited profibrotic effect in the absence of changes in aldosterone levels [53]. Importantly, high levels of MBG were associated with hypertension [33], stiffening of umbilical vessels, and elevated vascular level collagen-1 in PE patients, and *in vitro* incubation of the healthy blood vessels in the presence of low MBG concentration produced similar phenotype [41]. We hypothesized that aldosterone antagonists can also reverse MBG-induced vascular fibrosis, in the explants of the thoracic aorta and in the cultured rat vascular smooth muscle cells, and we observed that canrenone suppressed the effect of MBG synthesis of collagen-1 [23]. This observation was confirmed by clinical data which showed that in patients with resistant hypertension receiving spironolactone as an addition to the conventional antihypertensive therapy, there was a decrease of aortic vascular stiffness in parallel with an increase in the erythrocyte NKA [23].

Mineralocorticoid antagonists are known to reverse myocardial fibrosis [53]. It has been established that mineralocorticoid antagonists decrease blood pressure in rat hypertension models and suppress cardiac fibrosis, in which levels of CS including MBG are elevated [48, 49]. Thus, an interaction between Na/K-ATPase and MBG could be a target for aldosterone antagonists [53]. Considering the fact that MBG induces fibrosis in the cardiovascular system through the Fli-1-dependent mechanism, it was investigated the effects of spironolactone and its main metabolite, canrenone, for fibrosis in a series of experiments *in vitro* and *in vivo*. It has been shown that both spironolactone and canrenone weaken MBG-induced increase in collagen synthesis by myocardial fibroblasts, which was confirmed *in vivo* by a distinct decrease in cardiac fibrosis caused by experimental renal failure as a result of treatment with spironolactone [53]. As shown earlier, canrenone and spironolactone act as competing inhibitors of CS and NKA [50, 53–55]. Considering these, it can be assumed that short-term treatment with low doses of spironolactone or canrenone can be a very effective method of treating fibrotic manifestations of PE.

Spironolactone was introduced in 1960, and before 1980, this molecule was used for hypertension and PE [56, 57]. It was noted that when pregnant rats were treated with 40 mg spironolactone from 13 to 21 days of pregnancy, male foetuses showed signs of feminization [58]. Therefore, spironolactone is not advised for humans during pregnancy [59]. Considering that eplerenone has not been associated with adverse effects during pregnancy in animal studies, this drug is likely to be a better choice for use in pregnant women than spironolactone. However, no data on the use of these drugs in human pregnancy exist, and therefore, eplerenone should be used in pregnant women with heart failure only when treatment with other diuretics (such as furosemide) is noneffective [60].

Canrenone is the pharmacologically active metabolite of the spironolactone, used in hypertensive therapy. Canrenone exhibits its antialdosterone action because it blocks the binding of aldosterone to a cytosolic receptor in distal and collecting tubules of the nephron with subsequent inhibition of the synthesis of a specific protein that facilitates the entrance of Na^+^ ions into the cell and the consequent increment of NKA [61]. Several studies suggest that canrenone interacts with the ouabain-sensitive NKA competitively, by antagonizing the binding of ^3^H-ouabain [55] and MBG [53]. In addition to this well-known action, several studies have shown that canrenone was capable of inhibiting the NKA *in vitro* [50, 55, 62, 63]. Furthermore, if the pump was blocked by ouabain, canrenone could restimulate the pump [63, 64]. Interestingly, in experiment in rats, mineralocorticoid receptor antagonists also could reverse MBG-induced elevation of blood pressure associated with voluntary ethanol intake [65].

It has been shown that canrenone is a partial competitor of ouabain in the human placental membranes. Moreover, the analysis of the Scatchard plot shows a unique intercept suggesting that canrenone and ouabain compete for the same site [52]. The main findings of this study concern the effect of canrenone on the binding of ouabain to placental NKA and the observation that canrenone, at therapeutic concentrations (about 8 to 10 *μ*M), is able to reduce the ability of ouabain to inhibit the sodium pump [52]. Considering the above data, it can be suggested that therapeutic effects of canrenone can be a successful pathway in the treatment of PE due to its well-established antialdosterone action and to its modulatory effects on the NKA activity.

## 6. Conclusion

Currently, it is known that MBG is actively involved in the pathogenesis of the development of PE. Being an endogenous ligand of the NKA, MBG via the ionic and signaling pathways participates in the development of placental fibrosis, which may be the main pathological mechanisms of PE. We suggest that the system of MBG and the NKA is a keystone of PE pathogenesis. Immunoneutralization of MBG, as well as antagonism against binding with the NKA, can be a new and effective direction in the treatment of PreE and further prevention of its complications. In conclusion, our results indicate that in preeclampsia, elevated levels of MBG, via a Fli-1-dependent mechanism, stimulate synthesis of collagen in umbilical arteries, which leads to an impairment of vasorelaxation, and MBG-induced Fli-1-dependent signaling pathway contributes to vascular stiffness in preeclampsia. Recent studies have provided evidence that an excess of signaling molecules released by the placenta to circulation, soluble FMS-like tyrosine kinase-1 (sFlt1) and endoglin (sEng), or decreased levels of placental growth factor (PlGF) and vascular endothelial growth factor A (VEGF-A) play an important role in the pathogenesis of PE. Numerous candidate biomarkers have been proposed for prediction of PE and measurement of maternal circulating factors, such as ratio of sFlt-1/PlGF and reflected antiangiogenic balance that characterizes PE [66]. In our earlier work, in patients with PE levels of sFlt1 and of sEng were elevated together with CS [42], but whether or not both are interconnected remains to be understood. Interestingly, most of the studies of CS and of antiangiogenic balance did not make a distinction between early- and late-onset PE, which also represents an important area for future investigations.
